# Single-Cell Transcriptomic Analysis of Different Liver Fibrosis Models: Elucidating Molecular Distinctions and Commonalities

**DOI:** 10.3390/biomedicines13081788

**Published:** 2025-07-22

**Authors:** Guofei Deng, Xiaomei Liang, Yuxi Pan, Yusheng Luo, Zizhen Luo, Shaoxuan He, Shuai Huang, Zhaopeng Chen, Jiancheng Wang, Shuo Fang

**Affiliations:** 1 Scientific Research Center, The Seventh Affiliated Hospital of Sun Yat-sen University, Shenzhen 518107, China; denggf6@mail2.sysu.edu.cn (G.D.); liangxiaomei202@163.com (X.L.); panyx53@126.com (Y.P.); luoysh8@mail2.sysu.edu.cn (Y.L.); 2 Guangdong Provincial Key Laboratory of Digestive Cancer Research, The Seventh Affiliated Hospital of Sun Yat-sen University, Shenzhen 518107, China; 3 Medical Oncology, The Seventh Affiliated Hospital of Sun Yat-sen University, Shenzhen 518107, China; 4 School of Medicine, Sun Yat-sen University, Shenzhen 518107, China; luozzh3@mail2.sysu.edu.cn (Z.L.); heshx23@mail2.sysu.edu.cn (S.H.); huangsh226@mail2.sysu.edu.cn (S.H.); chenzhp76@mail2.sysu.edu.cn (Z.C.)

**Keywords:** liver fibrosis, immune microenvironment, single-cell RNA sequencing (scRNA-seq), animal models, molecular mechanisms

## Abstract

**Background**: Liver fibrosis, a consequence of various chronic liver diseases, is characterized by excessive accumulation of extracellular matrix (ECM), leading to impaired liver function and potentially progressing to cirrhosis or hepatocellular carcinoma. The molecular mechanisms underlying liver fibrosis are complex and not fully understood. In vivo experiments are essential for studying the molecular mechanisms of the disease. However, the diverse principles behind mouse modeling techniques for liver fibrosis can complicate the elucidation of specific fibrotic mechanisms. **Methods**: Five distinct liver fibrosis models were utilized: CONTROL, NASH (non-alcoholic steatohepatitis), BDL (bile duct ligation), TAA (thioacetamide), and CCl_4_ (carbon tetrachloride). Patents for these drugs were reviewed using Patentscope^®^ and Worldwide Espacenet^®^. ScRNA-seq was performed to analyze and compare the cellular and molecular differences in these models. **Results:** The analysis revealed that, particularly in the drug-induced fibrosis models, hepatic stellate cells (HSCs), Kupffer cells, and T-cell subsets exhibit distinct regulatory patterns and dynamic remodeling processes across different liver fibrosis models. These findings highlight the heterogeneity of immune responses and extracellular matrix (ECM) remodeling in various models, providing important insights into the complex mechanisms underlying liver fibrosis. **Conclusions**: The study enhances our understanding of liver fibrosis development and provides valuable insights for selecting the most representative animal models in future research. This comprehensive analysis underscores the importance of model-specific immune responses and ECM remodeling in liver fibrosis.

## 1. Introduction

Chronic liver injury and repair processes lead to the development of fibrosis, cirrhosis, and hepatocellular carcinoma (HCC) [1]. Hepatic fibrosis is characterized by the formation of fibrous scars due to the accumulation of extracellular matrix (ECM) proteins, primarily crosslinked type I and type III collagens, which replace the damaged normal tissue. Hepatic fibrosis is generally caused by two types of chronic liver injuries: hepatotoxic injury and cholestatic injury. The common causes of hepatic fibrosis can be further categorized into viral hepatitis, non-alcoholic steatohepatitis (NASH), alcoholic liver disease, genetic metabolic liver diseases, and cholestatic liver diseases [2,3]. Since over 80% of hepatocellular carcinoma occurs on the basis of hepatic fibrosis or cirrhosis, it poses a serious threat to public health [4]. A thorough exploration of the molecular mechanisms underlying hepatic fibrosis is crucial for developing effective prevention and treatment strategies.

To simulate the progression of diseases, various methods, such as chemical injury, genetic manipulation, and surgical operations, can be used to establish models [5]. Studies on patients with hepatic fibrosis and cirrhosis from different etiologies, along with experimental models of hepatic fibrosis in rodents, have revealed common key molecular mechanisms that contribute to liver fibrosis. Each method has its advantages and limitations, and the choice depends on specific research needs. For example, chemical-induced models like carbon tetrachloride (CCl_4_) [6] or thioacetamide (TAA) [7] are widely used due to their simplicity and reproducibility but may not fully capture the multifactorial nature of human liver fibrosis [8,9,10]. In contrast, genetic models, such as transgenic mice, can provide deep insights into the roles of specific molecular pathways in the development of fibrosis. However, genetic manipulation in these models may elicit non-specific effects that can affect the accuracy of research results [11].

The rapid advancements in bioinformatics have made large-scale data analysis possible, significantly propelling liver fibrosis disease research forward [12,13,14]. This study compares different animal models of liver fibrosis at the single-cell-sequencing level, revealing molecular characteristics within each model, including the heterogeneity of immune responses and ECM remodeling processes, which provides scientific guidance for the selection of animal models in liver fibrosis disease research.

## 2. Materials and Methods

### 2.1. Data Sources

The RNA sequencing (scRNA-Seq) data of 7 hepatocyte samples were downloaded from the GEO database (https://www.ncbi.nlm.nih.gov/geo/, accessed on 9 July 2024); these included 4 modeling groups: NASH, TAA, bile duct ligation (BDL), and CCl_4_, and the corn oil group was used as the control group (CONTROL). All samples were downloaded as raw files for subsequent analysis. The detailed information of the included GEO datasets is shown in Table S1.

### 2.2. Animal Models Included

Commonly used models of liver fibrosis include CCl_4_ administration, which induces liver injury and fibrosis through hepatocyte damage; BDL, which obstructs bile flow and leads to cholestatic liver injury; and thioacetamide (TAA) treatment, which causes liver damage via metabolic activation. Additionally, dietary models involving high-fat diets can induce non-alcoholic fatty liver disease (NAFLD) and non-alcoholic steatosis (NASH), while models utilizing chronic alcohol consumption replicate alcoholic liver disease [12,15,16]. These animal models provide crucial insights into the mechanisms of liver fibrosis and facilitate the evaluation of potential therapeutic interventions.

### 2.3. Data Loading, Quality Control, and Preprocessing

The raw single-cell RNA sequencing data, formatted according to 10× Genomics specifications, were processed using the CreateSeuratObject function from the Seurat package (version 4.3.0). The parameters were configured to exclude genes detected in fewer than five cells (min.cells = 5) and to retain cells expressing at least 300 genes (min.features = 300). For murine samples, the proportion of mitochondrial genes was calculated using the PercentageFeatureSet function with the pattern “^mt-”. Rigorous quality control was applied to filter out low-quality cells, retaining only those expressing between 200 and 5000 genes and exhibiting mitochondrial UMI ratios below 20%, utilizing the subset function. The filtered dataset was then subjected to log-normalization using the NormalizeData function with the “LogNormalize” method and a scale factor of 10,000. The top 2000 highly variable genes (HVGs) were identified using the FindVariableFeatures function with the “vst” selection method. Gene expression data were scaled to a mean of zero and a standard deviation of one using the ScaleData function while regressing out confounding effects attributable to mitochondrial content (percent.mt) and sequencing depth (nCount_RNA).

### 2.4. Dimensionality Reduction and Batch Integration

Principal component analysis (PCA) was conducted on scaled highly variable genes (HVGs) using the RunPCA function with the number of principal components (npcs) set to 50. The top 20 principal components (PCs), which accounted for at least 70% of the cumulative variance, as determined through elbow plot visualization, were selected for subsequent analyses [17]. In datasets containing multiple batches, batch effects were addressed using Harmony (version 0.1.1) via the RunHarmony function, with parameters set to group.by.vars = “batch”, reduction = “pca”, dims.use = 1:20, and max.iter.harmony = 10. The effectiveness of batch correction was assessed by comparing the embeddings before and after integration using DimPlot [18].

### 2.5. Cell Clustering and Visualization

A shared nearest neighbor (SNN) graph was constructed utilizing harmony-corrected embeddings for multi-batch data or principal component analysis (PCA) space for single-batch data (FindNeighbors, reduction set to “harmony” or “pca”; dimensions 1 through 20). Subsequently, graph-based clustering was executed using the Louvain algorithm (FindClusters, resolution set to 0.1). Cellular heterogeneity was visualized in two dimensions employing Uniform Manifold Approximation and Projection (UMAP) (RunUMAP, reduction set to “pca”, dimensions 1 through 20, with n.neighbors set to 30 and min.dist set to 0.3). The results were depicted in a DimPlot, with color coding reflecting either the cluster identity or experimental group.

### 2.6. Cell Type Annotation and Subpopulation Analysis

Cluster-specific marker genes were identified utilizing the FindAllMarkers function with rigorous criteria (only.pos = TRUE, min.pct = 0.25, logfc.threshold = 0.25, test.use = “wilcox”). Initial cell type annotation was conducted by cross-referencing the identified marker genes with established canonical cell type signatures. This annotation was validated through FeaturePlot, which assessed spatial expression, and VlnPlot, which examined distribution per cluster. To address cellular heterogeneity within major lineages, clusters corresponding to specific cell types were isolated using the subset function. These subpopulations underwent independent re-analysis, which included re-normalization, selection of highly variable genes (HVGs), principal component analysis (PCA) using the top 20 principal components, sub-clustering at a higher resolution (FindClusters, resolution = 0.05), UMAP projection, and subsequent marker gene identification. The marker genes used for cell type annotation are provided in Table S2.

### 2.7. Differential Expression and Gene Set Variation Analysis (GSVA)

Differentially expressed genes (DEGs) between the liver fibrosis model group and the control group were identified utilizing the FindMarkers function. The filtering criteria applied included a minimum detection fraction (min.pct) of 0.25, a minimum log_2_ fold-change (logfc.threshold) of 0.25, and the use of the “MAST” statistical test. DEGs were considered statistically significant if they had an adjusted *p*-value of less than 0.05. Subsequently, a Gene Set Variation Analysis (GSVA) was conducted on these significant DEGs to assess the enrichment of hallmark biological pathways [19]. The hallmark gene sets were accessed from the Molecular Signatures Database (MSigDB) (http://www.gsea-msigdb.org/gsea/index.jsp, accessed on 20 July 2024), using the R package “msigdbr”, specifying the species as “Mus musculus” and the category as “C5: GO Gene Sets” with the subcategory “GO: Biological Process”.

### 2.8. Pseudotime Trajectory Analysis

To reconstruct the temporal dynamics of cell populations and infer the progression of cellular states during differentiation or development, we conducted pseudotime trajectory analysis using the Monocle package (version 0.5.2) in R. This approach enabled us to order cells along a developmental timeline based on their transcriptomic profiles, even in the absence of explicitly measured time points.

### 2.9. Cell–Cell Communication Analysis

The CellChat package (available at https://github.com/sqjin/CellChat, accessed on 4 August 2024) was utilized to analyze cell–cell interactions among different cell types. This R package quantitatively calculates intercellular communication networks and predicts primary signaling pathways, which were subsequently used to visualize the signaling pathway networks [20]. Only ligands and receptors expressed in at least 10% of specific cells were considered for analysis.

### 2.10. Statistical Analysis

Statistical analyses were conducted using the R software (version 4.1.2) and GraphPad Prism (version 8.3.0). Data quantifications are reported as means ± standard deviation (SD). For comparisons among three or more groups, one-way ANOVA with Dunnett’s multiple comparisons test was used. For comparisons between two groups, a two-tailed Student’s *t*-test was applied. Statistical significance was assessed using a threshold of *p* < 0.05, with the following levels of significance indicated: * *p* < 0.05, ** *p* < 0.01, *** *p* < 0.001, and **** *p* < 0.0001.

## 3. Results

### 3.1. Integrating Single-Cell Data from Different Modeling Approaches

**Figure 1:**
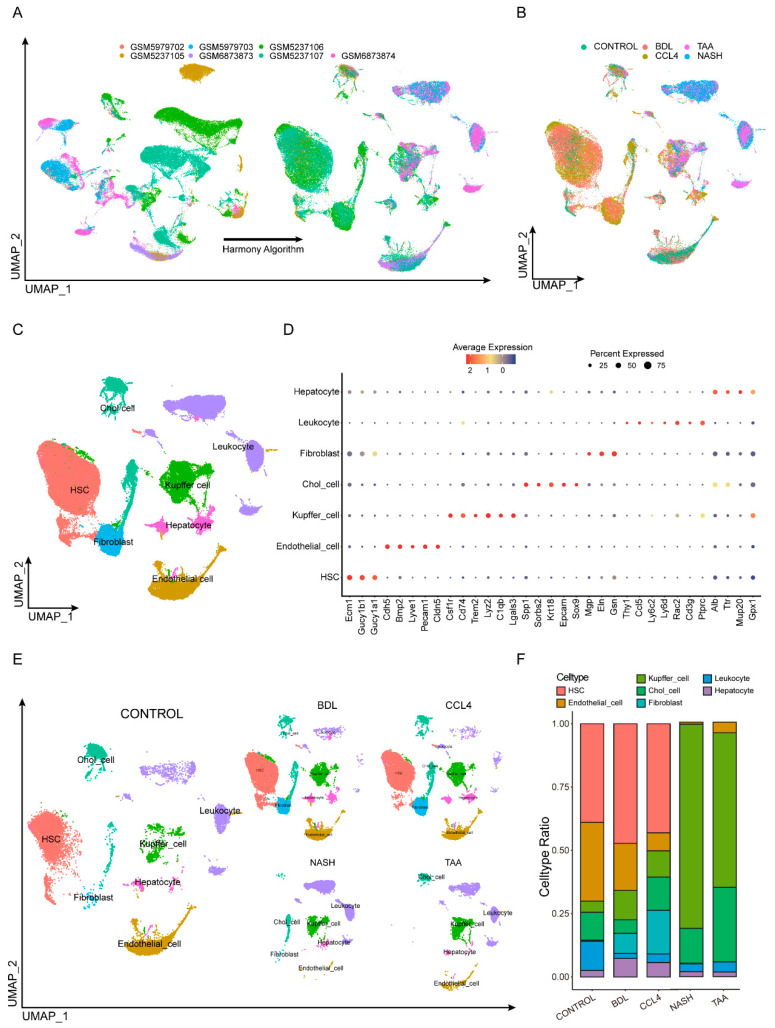
Integrating single-cell data from different modeling approaches. (**A,B**) Seven single-cell sequencing datasets were quality-controlled and filtered. (**C**) After harmony integration, these cells were clustered into 11 distinct clusters. (**D**) Markers of different cell types. (**E**) The distribution of cells varies depending on the type of modeling. (**F**) The prevalence of different cell types across all samples did not remain consistent.

To explore the heterogeneity in the liver fibrosis microenvironment across different experimental models, we consolidated several single-cell RNA sequencing datasets from murine models of liver fibrosis, including those induced by NASH, TAA, BDL, and CCl_4_. Additionally, single-cell data from normal murine livers were included as controls. A total of 13,605 cells met quality control standards. To mitigate batch effects across the distinct datasets, we applied the harmony algorithm for batch correction (Figure 1A,B). After harmony integration, these cells were clustered into 11 distinct clusters. Leveraging gene expression profiles characteristic of various cell types as described in the existing literature, we annotated these clusters into seven specific cell types [21]: Hepatic stellate cells (Ecm1, Gucy1b1, Gucy1a1), endothelial Cells (Cdh5, Bmp2, Lyve1, Pecam1, Cldn5), Kupffer cells (Csf1r, Cd74, Trem2, Lyz2, C1qb, Lgals3), cholangiocytes (Spp1, Sorbs2, Krt18, Epcam), fibroblasts (Sox9, Mgp, Eln, Gsn), leukocytes (Thy1, Ccl5, Ly6c2, Ly6d, Rac2, Cd3g, Ptprc), and hepatocytes (Alb, Ttr, Mup20, Gpx1), as illustrated in Figure 1C,D, and Table S3. The distribution of these seven cell types varies across different modeling types. For example, hepatic stellate cells (HSCs) dominate in the CONTROL, BDL, and CCl_4_ groups, while Kupffer cells are more prevalent in the NASH and TAA groups. This illustrates the heterogeneity of the microenvironment in different experimental models (Figure 1E,F).

### 3.2. HSC Differentiation Trajectories Across Different Fibrotic Models

**Figure 2:**
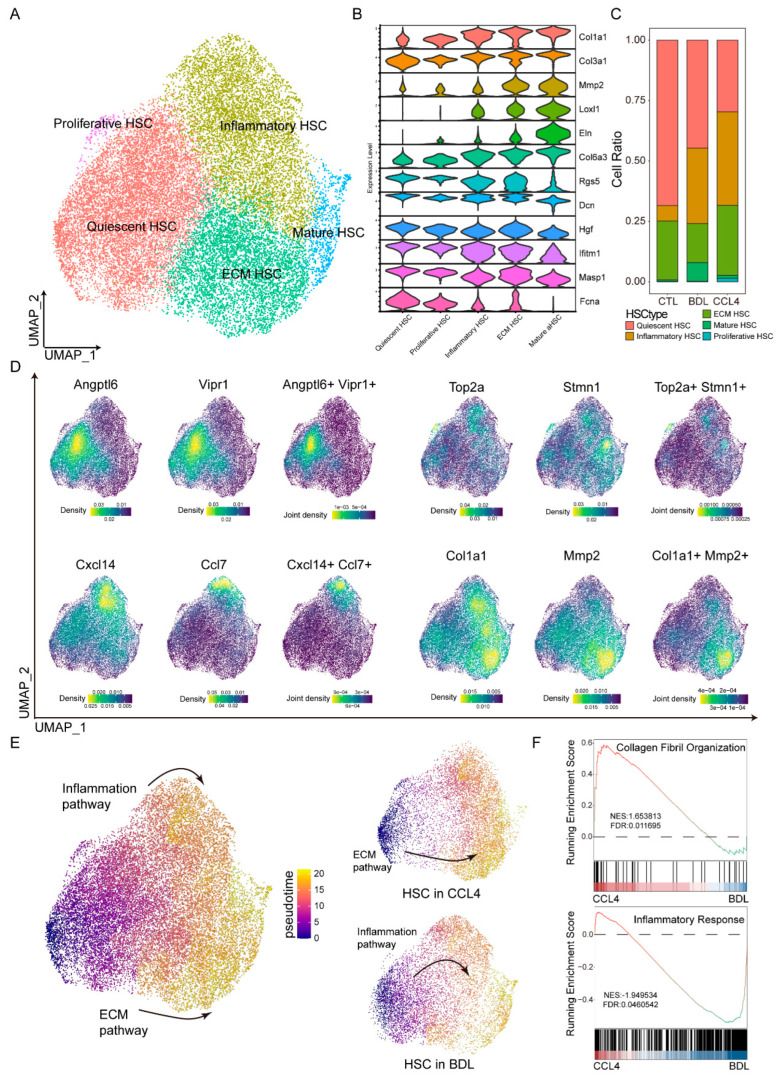
HSC differentiation trajectories across different fibrotic models. The UMAP plot demonstrates five distinct subpopulations of HSCs (**A**) identified through various markers (**D**). (**B**) Different subpopulations of HSCs are defined by the expression levels of extracellular matrix proteins. (**C**) Different modeling groups exhibit varying degrees of cell type enrichment. (**E**) Pseudotime analysis is utilized to determine the differentiation pathways of HSCs. (**F**) By utilizing Running Enrichment Scoring, the enrichment pathways of the CCl_4_ and BDL models are evaluated.

HSCs are pivotal drivers of liver fibrosis [22]. To dissect their heterogeneity during fibrogenesis, we subclustered HSCs and identified five distinct subtypes based on the expression of specific marker genes (Figure 2A,D). Specifically, we used marker genes characteristic of mature HSCs (Col1a1, Col3a1, Mmp2, Eln, Col6a3, Loxl1) and quiescent HSCs (Rgs5, Dcn, Hgf, Ifitm1, Masp1, Fcna), as defined by their expression patterns along the activation trajectory [23] (Figure 2B and Figure S1). The distribution of these HSC subtypes varied substantially across different liver fibrosis models (Figure 2C). Consistent with the pseudotime trajectory analysis, which revealed distinct activation phenotypes (Figure 2E), we found that HSCs in the CCl_4_ model were predominantly associated with an extracellular matrix remodeling phenotype, whereas HSCs in the TAA model exhibited a preference for an immune-activated phenotype. Functional analysis further supported these phenotypic differences: in the CCl_4_; model, enhanced signaling related to collagen fibril organization and response to mechanical stimuli highlighted the predominance of ECM remodeling. Conversely, in the TAA model, increased activity of inflammatory pathways indicated a tendency toward immune-related activation (Figure 2F).

### 3.3. Heterogeneity of Kupffer Cells in Liver Fibrosis Models

**Figure 3:**
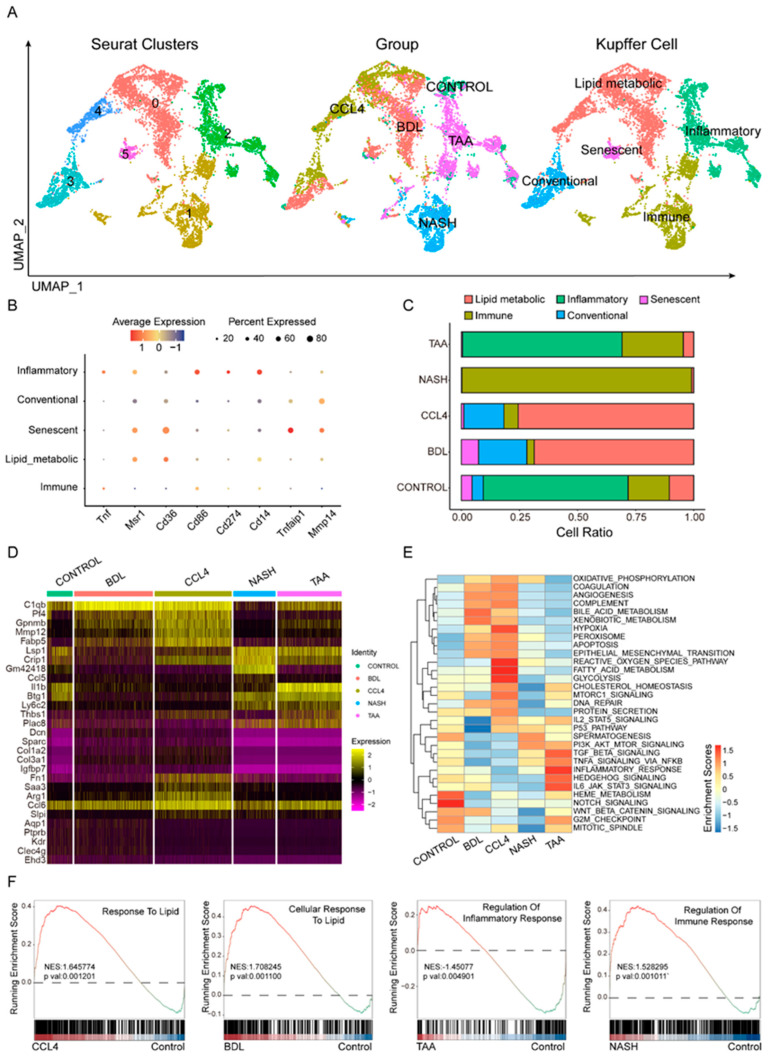
Heterogeneity of Kupffer cells in liver fibrosis models. Using eight cellular markers (**B**), the primary types of Kupffer cells in different fibrosis modeling groups are defined (**A**). (**C**) Comparison of proportions of different Kupffer cell subtypes among various fibrosis modeling groups. Different modeling groups exhibit differential genes (**D**) and enriched pathways (**E**). (**F**) Pathway enrichment results of different modeling methods are shown.

Kupffer cells are essential in the progression of liver fibrosis [24,25,26]. Following quality control and screening using Seurat, single-cell RNA sequencing (scRNA-seq) was employed to analyze Kupffer cells across various hepatic fibrosis models, including CONTROL, BDL, CCl_4_, NASH, and TAA, to investigate cellular heterogeneity (Figure 3A). Markers such as Tnf, Msr1, Cd36, Cd86, Cd274, Cd14, Tnfaip1, and Mmp14 were utilized to distinguish different Kupffer cell subtypes (Figure 3B, Table S4), which were categorized into five distinct subsets: inflammatory, lipid metabolic, senescent, conventional, and immune response. The distribution of these Kupffer cell subtypes varied markedly among the different fibrosis models (Figure 3C). In the NASH model, immune-related subtypes predominated. Both the CCl_4_ and BDL models were characterized primarily by lipid metabolism-related subtypes. Conversely, the TAA model resembled the control group, with inflammation-related subtypes being predominant. Differential gene analysis (Figure 3D) identified signature genes specific to each of the four liver fibrosis models. A metabolic pathway enrichment analysis revealed that distinct metabolic pathways were enriched in the different hepatic fibrosis models, encompassing fatty acid metabolism, glycolysis, hypoxia, bile acid metabolism, and inflammatory response (Figure 3E). The GSVA analysis results indicated that, compared to the control group, the CCl_4_ model showed enrichment in fatty acid metabolism-related pathways, suggesting a propensity for lipid metabolism-related differentiation. The BDL model exhibited high expression in the bile acid metabolic pathway, indicating a preference for differentiation pathways related to lipid metabolism. The TAA models displayed enrichment in inflammatory pathways, suggesting a tendency towards differentiation pathways that regulate inflammatory responses. In the NASH models, high expression of the PI3K-AKT signaling pathway implied a propensity for differentiation pathways involved in the regulation of immune responses (Figure 3F).

### 3.4. Dynamic T-Cell Subsets in Liver Fibrosis

**Figure 4:**
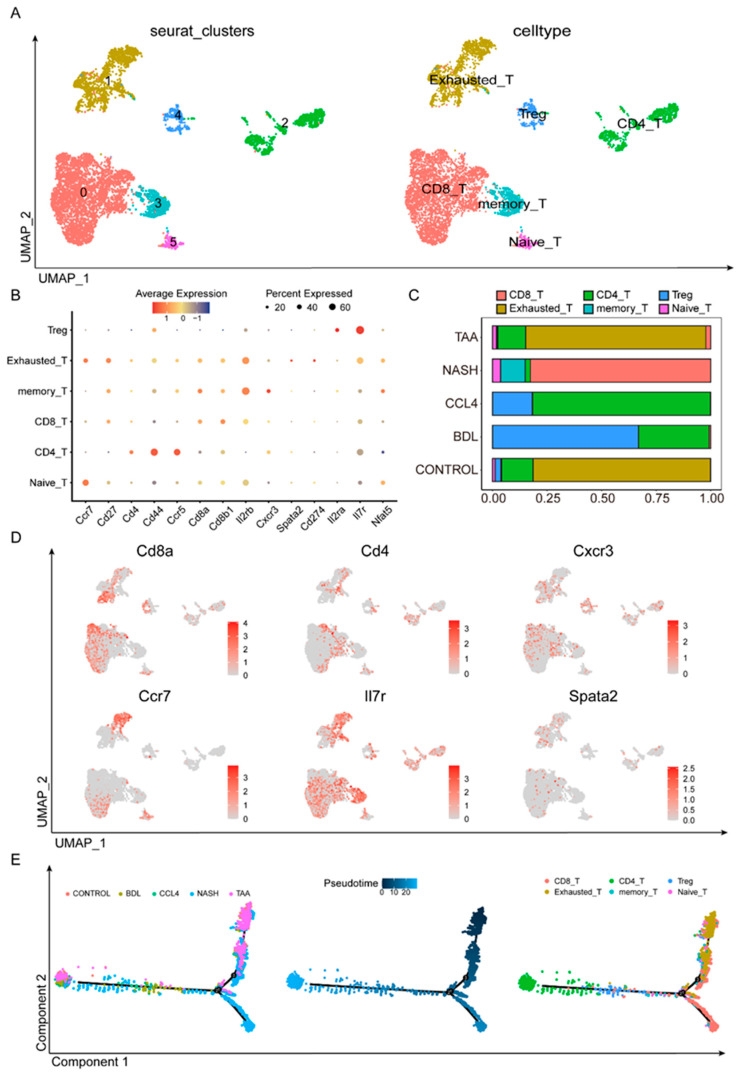
Dynamic T-cell subsets in liver fibrosis. Using 14 cellular markers (**B**), the primary types of T cells in different fibrosis modeling groups are defined (**A**). (**C**) The proportion of T-cell subsets in different liver fibrosis modeling methods. (**D**) Using various markers, the expression levels of different T-cell subpopulations in various modeling methods are observed. (**E**) Pseudotime analysis found that different modeling methods had different differentiation pathways.

T cells are pivotal in adaptive immune responses, and their subpopulations were further delineated through the analysis of scRNA-seq data. Following Seurat-based quality control and filtering, we examined the T-cell subgroups (Figure 4A). Various T-cell subpopulations, including Treg, exhausted T cells, memory T cells, CD8 T cells, CD4 T cells, and naïve T cells, were identified using a set of cell markers (Ccr7, Cd27, CD4, Cd44, Ccr5, Cd8a, Cd8b1, etc.) (Figure 4B,D, Table S5). Notably, the composition of T-cell subpopulations differed significantly across the different modeling methods (Figure 4C). TAA was similar to CONTROL, where exhausted T cells played a central role. In the NASH group, CD8 T cells were predominant. In both the CCl_4_ and BDL groups, CD4 T cells and Treg cells were prominent. However, the CCl_4_ group had a higher proportion of CD4 T cells, whereas BDL was characterized primarily by Treg cells. The pseudotime analysis results (Figure 4E) indicated two distinct differentiation pathways within the T-cell subsets. In the liver fibrosis models, non-effector cells like naïve T cells, Tregs, and exhausted T cells progressively differentiated into effector T-cell pathways, including CD4, CD8, and memory T cells. Among these, CD8 cells were predominant in the NASH group, exhausted T cells were dominant in the TAA group, and the CD4 cell differentiation pathway was prominent in the BDL and CCl_4_ groups. This suggests that BDL, CCl_4_, and NASH primarily exerted their effects through the effector T-cell pathway, while the TAA group might play a distinct role in liver fibrosis development via other non-immune pathways.

### 3.5. Cell–Cell Interaction Networks in Liver Fibrosis

**Figure 5:**
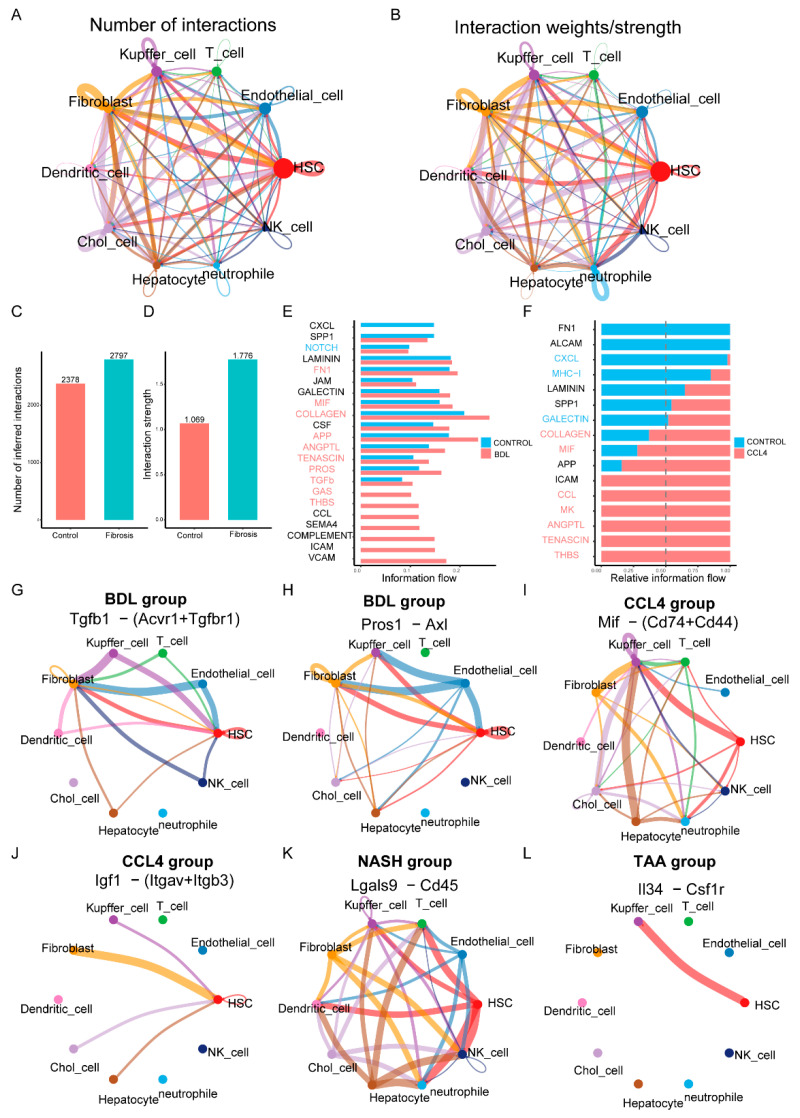
Cell–cell interaction networks in liver fibrosis. (**A**,**B**) The number of interactions and the interaction weights/strength of each cell. (**C**,**D**) The analysis showed a significant increase in the number and strength of interactions between hepatocytes, hematopoietic stem cells, Kupffer cells, and other stromal cells such as fibroblasts and endothelial cells in the liver fibrosis modeling group compared to the control group. (**E**,**F**) Differential gene expression in CCl_4_ and BDL fabrication and control group. (**G**–**L**) Receptor–ligand pairs that are characteristically expressed in different manufacturing modules.

To elucidate the interactions between different cell types in liver fibrosis, we constructed a cell-to-cell interaction network (Figure 5A,B). The analysis revealed a substantial increase in the number and intensity of interactions between hepatocytes, HSCs, Kupffer cells, and other stromal cells, such as fibroblasts and endothelial cells, in the liver fibrosis modeling group compared to the control group (Figure 5C,D). Notably, in the different modeling groups, there were close interactions between HSCs and various other cells, which may reflect the activation of HSCs during fibrosis. By integrating data from different models, we created a comprehensive map of signaling pathways and interaction networks. These networks not only revealed key nodes of cell communication but also highlighted potential therapeutic targets during liver fibrosis. For instance, the information flow of molecules such as VCAM, ICAM, SEMA4, GAS, COLLAGEN, and GALECTIN changed significantly in the BDL modeling group compared to the CONTROL group (Figure 5E). Similarly, the expression levels of THBS, TENASCIN, ANGPTL, MK, and MIF in the CCl_4_ modeling group were notably different from those in the control group (Figure 5F). This might indicate their critical role in the progression of liver fibrosis. Further analysis confirmed cell type-specific molecular expression patterns in different models. For example, the tgfβ1-(Acvr1+Tgfbr1) and pros1-axl receptor–ligand pairs were upregulated in the BDL model (Figure 5G,H). In CCl_4_-induced models, the expression of Mif-(Cd74+Cd44) and Igf1-(Itgav+Itgb3) receptor–ligand pairs increased (Figure 5I,J). Additionally, the Lgals9-Cd45 (Figure 5K) and Il34-Csf1r (Figure 5L) receptor–ligand pairs were significantly upregulated in models induced by NASH and TAA, respectively. These results suggest that different modalities of liver fibrosis modeling might function through different receptor–ligand pairs.

## 4. Discussion

Liver fibrosis is a common intermediate stage of chronic liver disease, characterized by the excessive deposition of extracellular matrix (ECM), which significantly impacts the progression of various liver diseases [26,27]. To deepen our understanding of the progression of liver diseases, it is crucial to comprehend their pathophysiological mechanisms. The advent of single-cell RNA sequencing (scRNA-seq) has revolutionized our understanding of the heterogeneity of cellular responses in liver fibrosis, offering insights into molecular complexities that were previously obscured in bulk RNA sequencing studies [28,29]. Our study utilized scRNA-seq to dissect the cellular and molecular landscape of liver fibrosis induced by different modeling methods, including CONTROL, BDL, CCl_4_, NASH, and TAA. By integrating scRNA-Seq data from multiple liver cell types, we revealed distinct molecular characteristics and cellular dynamics that reflect the unique patterns of multifactorial etiology in human liver disease. Through the identification of cell marker expression levels, we distinguished the cell types that play a critical role in the process of liver fibrosis, including HSCs, T cells, and Kupffer cells. Additionally, we employed differential gene analysis to observe the heterogeneity of HSCs, Kupffer cells, and T cells across different models, highlighting the complex interplay of immune responses, ECM remodeling, and cellular differentiation pathways in the fibrotic process.

**Figure 6:**
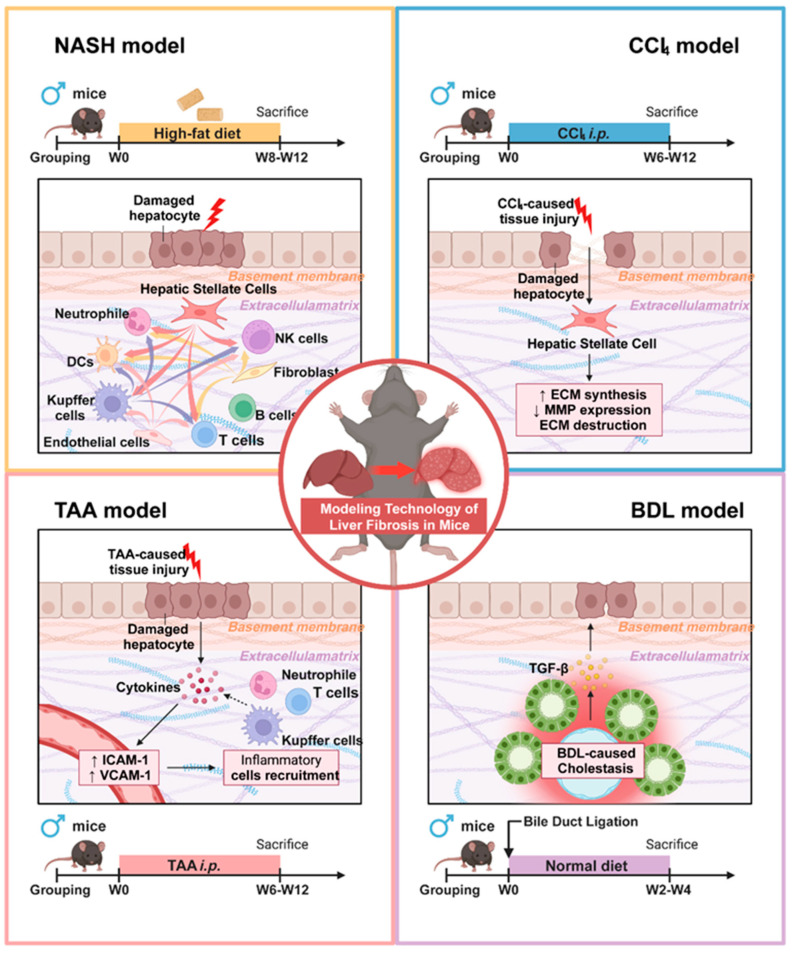
Pathogenic mechanisms and molecular distinctions among different liver fibrosis modeling approaches.

The current study has revealed that HSCs play a central role in the process of liver fibrosis, not only by producing collagen, fibronectin, and other ECM components that accumulate in the extracellular matrix, leading to structural changes and functional impairment of the liver, but also by participating in inflammatory responses, immune modulation, intercellular communication, metabolic level, and ECM remodeling processes [30,31]. The study by Yang et al. demonstrates that in a CCl_4_-induced liver fibrosis model, HSCs are the primary contributors to myofibroblast formation. These cells sequentially activate inflammatory, migratory, and ECM production pathways, resulting in substantial collagen secretion. This process leads to excessive ECM deposition and increased collagen fiber cross-linking, which directly enhances the mechanical stiffness of the liver matrix. The increased stiffness further stimulates HSC activation through a positive feedback mechanism, thereby intensifying ECM accumulation. In contrast, BDL modeling induces liver fibrosis via bile duct obstruction, causing bile stasis and subsequent liver fibrosis. In this model, HSCs become the predominant source of ECM. Metabolic disturbances, such as imbalances in bile acid metabolism, promote ECM secretion by HSCs and simultaneously disrupt ECM metabolic homeostasis, resulting in abnormal deposition. Zhang et al.’s research showed that during TAA modeling, the metabolites induce hepatocyte damage, leading to the release of injury-associated molecular patterns that activate inflammatory signaling pathways and recruit neutrophils. While the reactive oxygen species and proteases released by neutrophils primarily exacerbate hepatocellular injury, they also indirectly facilitate abnormal ECM deposition and remodeling within the inflammatory microenvironment. In the context of NASH modeling, CD3^+^, CD4^+^, CD8^+^ T cells, and B cells congregate to form lymphoid follicles. Macrophages engulf lipids, transforming into foam cells and forming granulomatous structures with lymphocytes. These immune cells secrete cytokines such as IL-6 and TNF-α, which stimulate HSCs to activate and enhance ECM synthesis, thereby leading to more pronounced ECM deposition in the inflamed regions (Figure 6 and Table S6).

In this study, we not only identified the distinct molecular characteristics of various HSC subgroups but also elucidated their potential associations with the underlying mechanisms in different drug-induced liver fibrosis models. Specifically, in the carbon tetrachloride CCl_4_ model, HSC subgroups were predominantly enriched in ECM-related pathways, whereas in the BDL model, these subgroups were primarily associated with inflammatory pathways. This discovery further delineates the different roles of HSCs in various models [32,33,34]. Additionally, we conducted a comprehensive analysis of immune cells that significantly contribute to liver fibrosis. A differential gene analysis of Kupffer cell subgroups indicated that only the CCl_4_ and BDL models exhibited similar proportions of Kupffer cell subtypes closely linked to lipid metabolism, suggesting distinct roles for Kupffer cells in the progression of liver fibrosis across different etiologies. Furthermore, analysis of T-cell subgroups revealed a significant increase in the proportion of exhausted T cells in liver fibrosis models compared to the control group, aligning with emerging research that identifies immune exhaustion as a hallmark of chronic liver disease. This provides a new perspective on the mechanisms of disease tolerance and progression.

Moreover, the intercellular interaction network we have developed offers comprehensive maps of signaling pathways and interaction networks, elucidating key nodes that may serve as therapeutic targets in cell communication. This includes notable alterations in the molecular information flow of molecules such as VCAM, ICAM, and SEMA4 within the BDL model. Recent studies have corroborated their potential roles in the fibrosis process, underscoring the importance of selecting appropriate models for mechanistic research and therapeutic exploration [35,36]. These findings align with current research conclusions, highlighting the role of the extracellular matrix (ECM) in liver fibrosis and the influence of the inflammatory microenvironment on disease progression. Furthermore, these studies have enhanced our understanding of the multi-dimensional mechanisms underlying liver fibrosis by investigating the molecular characteristics of cell subpopulations, the functional differences among immune cells, and the regulatory networks between cells.

In conclusion, this study underscores the intricate nature of liver fibrosis pathology and underscores the utility of scRNA-seq in unraveling the cellular and molecular underpinnings of the disease. Building upon the foundational research utilizing the CCl_4_, BDL, TAA, and NASH models, our findings not only refine the molecular distinctions among HSC subgroups across various etiologies of liver fibrosis but also offer a novel, multi-dimensional perspective on the mechanisms and therapeutic exploration of liver fibrosis. This is achieved through the analysis of the etiological specificity of Kupffer within the intercellular communication network. These insights provide a scientific framework for future investigations aimed at selecting appropriate models and identifying potential therapeutic targets.

There are some limitations to this study. Firstly, this study focused on the application of HSCs and immune cells in the context of liver fibrosis, but the functions of non-immune cells were not explored. Secondly, we did not verify the genes and signaling pathways that played a key role in different modeling methods through in vitro or in vivo experiments. Thirdly, we included only seven relevant single-cell sequencing datasets, which represent a relatively small sample size. Further research is needed to validate these findings in larger single-cell sequencing datasets and to verify the critical roles of relevant genes and signaling pathways through experimental validation.

## 5. Conclusions

In summary, our study highlights the complexity of liver fibrosis pathology and underscores the value of scRNA-seq in elucidating the cellular and molecular basis of the disease. The results offer a detailed perspective on the differences in cellular responses and gene expression among various liver fibrosis modeling methods, providing scientific guidance for the selection of appropriate models and potential therapeutic targets in future research.

## Data Availability

All datasets analyzed in this study are publicly available in the NCBI GEO repository (accession numbers: GSE221481, GSE199638, GSE171904). Links to the datasets are provided in Section 2 and Supplementary File. Analysis code (R scripts) is available at: https://github.com/Gofree0504/Single-cell-RNA-seq-Data-Processing-Pipeline (accessed on 17 July 2025).

## Supplementary Materials

The following supporting information can be downloaded at: https://www.mdpi.com/article/10.3390/biomedicines13081788/s1, Figure S1: Differential Expression of Extracellular Matrix Proteins Characterizes Distinct HSC Subpopulations in Various Groups; Table S1: The detailed information of the included GEO datasets; Table S2: Marker genes applied in cell annotation; Table S3: The clustering basis and characteristic gene functions of hepatic stellate cells (HSCs) and fibroblasts; Table S4: Gene Lists for Pathways of Kupffer cells in Figure 3A; Table S5: explicit and detailed definitions for each T cell subset; Table S6: Specific mechanisms and application scenarios of different liver fibrosis modeling methods.
