# Supplementary material for: Single-Cell Transcriptomic Analysis of Different Liver Fibrosis Models: Elucidating Molecular Distinctions and Commonalities
Source: Biomedicines. 2025 Jul 22;13(8):1788. doi: 10.3390/biomedicines13081788 (PMC12383845; doi:10.3390/biomedicines13081788)
Supplement: Supplementary file 1 [file biomedicines-13-01788-s001.zip › biomedicines-3744035-supplementary.pdf]

Supplemental material

Single-Cell Transcriptomic Analysis of Different Liver Fibrosis Models: Elucidating Molecular Distinctions and Commonalities

Guofei Deng#, Xiaomei Liang#, Yuxi Pan#, Yusheng Luo, Zizhen Luo, Shaoxuan He, Shuai Huang, Zhaopeng Chen, Jiancheng Wang*, Shuo Fang*

Content

Supplementary Figures (Figure S1)

Supplementary Tables (Table S1-S6)

Supplementary Figure S1. Differential Expression of Extracellular Matrix Proteins Characterizes Distinct HSC Subpopulations in Various Groups.

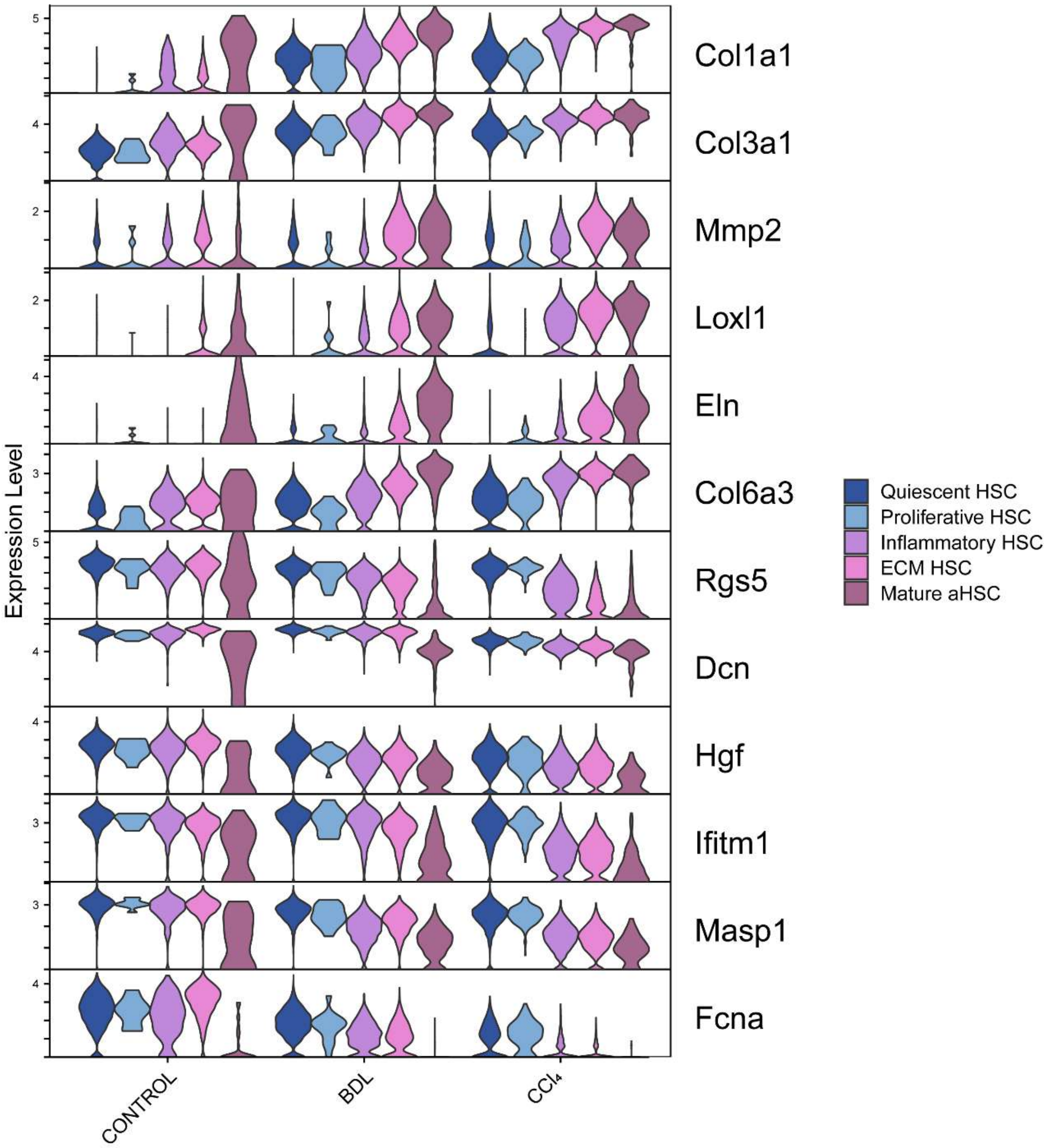

Supplementary TableS1. **The detailed information of the included GEO datasets.**

Series	type	number	species	number of cells	PMID
GSE221481	TAA	2	Mus musculus	25339	38010435
GSE199638	NASH	2	Mus musculus	24144	37756381
GSE171904	CCl_4/BDL	3	Mus musculus	64928	34089528

Supplementary Table S2. **Marker genes applied in cell annotation.**

Cell type	Marker	Reference
Hepatic stellate cells	Ecm1	Single-Cell Transcriptomics Uncovers Zonation of Function in the Mesenchyme during Liver Fibrosis. ***Cell Reports***, 29(7), 1832–1847.e8.
	Gucy1b1<br>Gucy1a1	Single-cell transcriptomic analysis reveals a hepatic stellate cell–activation roadmap and myofibroblast origin during liver fibrosis in mice. ***J Hepatol***. 2021;75(5):1203–1214. doi: 10.1016/j.jhep.2021.06.032
Endothelial Cells	Cdh5	A molecular atlas of cell types and zonation in the brain vasculature. ***Nature***. 2018 Feb 22;554(7693):475-480. doi: 10.1038/nature25739. Epub 2018 Feb 14. Erratum in: Nature. 2018 Aug;560(7716): E3. doi: 10.1038/s41586-018-0232-x. PMID: 29443965.
	Bmp2	Molecular and Spatial Signatures of Mouse Embryonic Endothelial Cells at Single-Cell Resolution. ***Circ Res.*** 2024 Mar;134(5):529-546. doi: 10.1161/CIRCRESAHA.123.323956. Epub 2024 Feb 13. PMID: 38348657; PMCID: PMC10906678.
	Lyve1	Defining murine organogenesis at single-cell resolution reveals a role for the leukotriene pathway in regulating blood progenitor formation. ***Nat Cell Biol.*** 2018 Feb;20(2):127-134. doi: 10.1038/s41556-017-0013-z. Epub

		2018 Jan 8. PMID: 29311656; PMCID: PMC5787369.
	Pecam1	A molecular atlas of cell types and zonation in the brain vasculature. ***Nature***. 2018 Feb 22;554(7693):475-480. doi: 10.1038/nature25739. Epub 2018 Feb 14. Erratum in: Nature. 2018 Aug;560(7716):E3. doi: 10.1038/s41586-018-0232-x. PMID: 29443965.
	Cldn5	Claudin5 protects the peripheral endothelial barrier in an organ and vessel-type-specific manner. ***Elife.*** 2022 Jul 21;11: e78517. doi: 10.7554/eLife.78517. PMID: 35861713; PMCID: PMC9348850.
Kupffer Cells	Csf1r<br>Cd74<br>Lyz2<br>Trem2<br>C1qb<br>Lgals3	Single-cell RNA sequencing reveals a novel inhibitory effect of ApoA4 on NAFL mediated by liver-specific subsets of myeloid cells. ***Front Immunol.*** 2022 Nov 8; 13:1038401. doi: 10.3389/fimmu.2022.1038401. PMID: 36426356; PMCID: PMC9678944.
Cholangiocytes	Spp1<br>Sorbs2<br>Krt18<br>Epcam	Single-cell RNA-Seq analysis reveals dynamic trajectories during mouse liver development. ***BMC Genomics***. 2017 Dec 4;18(1):946. doi: 10.1186/s12864-017-4342-x. PMID: 29202695; PMCID: PMC5715535.
Fibroblasts	Sox9	SOX9: a key transcriptional regulator in organ fibrosis. ***Front Pharmacol.*** 2025 Feb 5; 16:1507282. doi: 10.3389/fphar.2025.1507282. PMID: 39974732; PMCID: PMC11835943.
	Mgp<br>Eln<br>Gsn	Single-Cell Transcriptomic Analysis Reveals a Hepatic Stellate Cell-Activation Roadmap and Myofibroblast Origin During Liver Fibrosis in Mice. ***Hepatology***. 2021 Nov;74(5):2774-2790.

		doi: 10.1002/hep.31987. Epub 2021 Aug 21. PMID: 34089528; PMCID: PMC8597108.
Leukocytes	Thy1<br>Ccl5<br>Ly6c2<br>Ly6d<br>Rac2<br>Cd3g<br>Ptprc	Single-Cell Transcriptomic Analysis Reveals a Hepatic Stellate Cell-Activation Roadmap and Myofibroblast Origin During Liver Fibrosis in Mice. ***Hepatology***. 2021 Nov;74(5):2774-2790. doi: 10.1002/hep.31987. Epub 2021 Aug 21. PMID: 34089528; PMCID: PMC8597108.
Hepatocytes	Alb<br>Ttr<br>Mup20<br>Gpx1	Single-Cell Transcriptomic Analysis Reveals a Hepatic Stellate Cell-Activation Roadmap and Myofibroblast Origin During Liver Fibrosis in Mice. ***Hepatology***. 2021 Nov;74(5):2774-2790. doi: 10.1002/hep.31987. Epub 2021 Aug 21. PMID: 34089528; PMCID: PMC8597108

Supplementary Table S3. The clustering basis and characteristic gene functions of hepatic stellate cells (HSCs) and fibroblasts.

Gene	Cell type	Reference
Ecm1	HSCs	ECM1 attenuates hepatic fibrosis by interfering with mediators of latent TGF-β1 activation. ***Gut***. 2025 Feb 6;74(3):424-439. doi: 10.1136/gutjnl-2024-333213. PMID: 39448254.
Gucy1b1	HSCs	Single-Cell Transcriptomic Analysis Reveals a Hepatic Stellate Cell-Activation Roadmap and Myofibroblast Origin During Liver Fibrosis in Mice. ***Hepatology***. 2021 Nov;74(5):2774-2790. doi: 10.1002/hep.31987. Epub 2021 Aug 21. PMID: 34089528; PMCID: PMC8597108.
Gucy1a1	HSCs	
Mgp	Fibroblasts	CD201+ fascia progenitors choreograph injury repair. ***Nature***. 2023 Nov;623(7988):792-802. doi: 10.1038/s41586-023-06725-x. Epub 2023 Nov 15. Erratum in: Nature. 2024 Jan;625(7993): E4. doi: 10.1038/s41586-023-06928-2. PMID: 37968392; PMCID: PMC10665192.
Eln	Fibroblasts	ALKBH5-mediated mA demethylation ameliorates extracellular matrix deposition in cutaneous pathological fibrosis. ***Clin Transl Med***. 2024 Sep;14(9): e70016. doi: 10.1002/ctm2.70016. PMID: 39233335; PMCID: PMC11374695.
Gsn	Fibroblasts	Gelsolin's Protective Role in MASH through F-Actin Regulation and P53 Degradation. ***Adv Sci (Weinh)***. 2025 Jun;12(23): e2416489. doi: 10.1002/advs.202416489. Epub 2025 May 20. PMID: 40390546; PMCID: PMC12199436.

Supplementary Table S4. Gene Lists for Pathways of Kupffer cells in Fig 3A.

Functional Pathway	Marker Genes	Reference
Inflammatory	Tnf	Tumour necrosis factor alpha signalling through activation of Kupffer cells plays an essential role in liver fibrosis of non-alcoholic steatohepatitis in mice. ***Gut.*** 2006 Mar;55(3):415-24. doi: 10.1136/gut.2005.071118. Epub 2005 Sep 20. PMID: 16174657; PMCID: PMC1856073.
	Nos2	Impaired macrophage autophagy increases the immune response in obese mice by promoting proinflammatory macrophage polarization. ***Autophagy***. 2015;11(2):271-84. doi: 10.1080/15548627.2015.1009787. PMID: 25650776; PMCID: PMC4502775.
	Ly6C1	Macrophage heterogeneity in liver injury and fibrosis. ***J Hepatol.*** 2014 May;60(5):1090-6. doi: 10.1016/j.jhep.2013.12.025. Epub 2014 Jan 8. PMID: 24412603.
Conventional	Cd14	Activation of human and mouse Kupffer cells by lipopolysaccharide is mediated by CD14. ***Am J Physiol Gastrointest Liver Physiol.*** 2002 Sep;283(3):G640-5. doi: 10.1152/ajpgi.00253.2001. PMID: 12181178.
	Tim4	T-Cell Immunoglobulin and Mucin Domain-Containing Protein-4 Is Critical for Kupffer Cell Homeostatic Function in the Activation and Resolution of Liver Ischemia Reperfusion Injury. ***Hepatology.*** 2021 Oct;74(4):2118-2132. doi: 10.1002/hep.31906. Epub 2021 Aug 10. PMID: 33999437; PMCID: PMC9060306.
	Mmp14	Expression patterns of matrix metalloproteinases and their inhibitors in parenchymal and non-parenchymal cells of rat liver: regulation by TNF-

		alpha and TGF-beta1. ***J Hepatol.*** 1999 Jan;30(1):48-60. doi: 10.1016/s0168-8278(99)80007-5. PMID: 9927150.
	Ly75	CD205-TLR9-IL-12 axis contributes to CpG-induced oversensitive liver injury in HBsAg transgenic mice by promoting the interaction of NKT cells with Kupffer cells. ***Cell Mol Immunol.*** 2017 Aug;14(8):675-684. doi: 10.1038/cmi.2015.111. Epub 2016 Apr 4. PMID: 27041637; PMCID: PMC5549602.
Senescent	Waf1	Fetal origin confers radioresistance on liver macrophages via p21. ***J Hepatol.*** 2019 Sep;71(3):553-562. doi: 10.1016/j.jhep.2019.04.015. Epub 2019 May 9. PMID: 31077791.
	Tnfaip1	TNFAIP1 promotes macrophage lipid accumulation and accelerates the development of atherosclerosis through the LEENE/FoxO1/ABCA1 pathway. ***J Physiol Biochem.*** 2024 Aug;80(3):523-539. doi: 10.1007/s13105-024-01018-x. Epub 2024 Jun 15. PMID: 38878215.
Lipid metabolic	Cd36	A subset of Kupffer cells regulates metabolism through the expression of CD36. ***Immunity.*** 2021 Sep 14;54(9):2101-2116.e6. doi: 10.1016/j.immuni.2021.08.006. Epub 2021 Aug 31. PMID: 34469775.
	Msr1	Macrophage scavenger receptor 1 mediates lipid-induced inflammation in non-alcoholic fatty liver disease. ***J Hepatol.*** 2022 May;76(5):1001-1012. doi: 10.1016/j.jhep.2021.12.012. Epub 2021 Dec 21. PMID: 34942286.
	Scara1	Kupffer cell programming by maternal obesity triggers fatty liver disease. ***Nature.*** 2025 Jun 18. doi: 10.1038/s41586-025-09190-w. Epub ahead of print. PMID: 40533564.
Immune-regulatory	Cd86	Single cell RNA sequencing of human liver reveals distinct intrahepatic macrophage populations. ***Nat***

		Commun. 2018 Oct 22;9(1):4383. doi: 10.1038/s41467-018-06318-7. PMID: 30348985; PMCID: PMC6197289.
	Cd274	PD-1 blockade improves Kupffer cell bacterial clearance in acute liver injury. ***J Clin Invest.*** 2021 Feb 15;131(4): e140196. doi: 10.1172/JCI140196. PMID: 33320839; PMCID: PMC7880414.
	Cd80	Identification of a Kupffer cell subset capable of reverting the T cell dysfunction induced by hepatocellular priming. ***Immunity.*** 2021 Sep 14;54(9):2089-2100.e8. doi: 10.1016/j.immuni.2021.05.005. Epub 2021 Aug 31. PMID: 34469774; PMCID: PMC8459394.
	H2	Kupffer cells determine intrahepatic traffic of PEGylated liposomal doxorubicin. ***Nat Commun.*** 2024 Jul 20;15(1):6136. doi: 10.1038/s41467-024-50568-7. PMID: 39033145; PMCID: PMC11271521.
	Cd40	A liver-centric help circuit revives CD8 T cells via IL-27. ***Nat Immunol.*** 2025 Jul 8. doi: 10.1038/s41590-025-02204-9. Epub ahead of print. PMID: 40629127.

Supplementary TableS5. **explicit and detailed definitions for each T cell subset.**

Pathways	Marker	Reference
CD8+T cell	Cd8a, Cd8b1, Spata2	Spatial heterogeneity of infiltrating T cells in high-grade serous ovarian cancer revealed by multi-omics analysis. ***Cell Rep Med.*** 2022 Dec 20;3(12):100856. doi: 10.1016/j.xcrm.2022.100856. PMID: 36543113; PMCID: PMC9798026. Integrated single-cell profiling dissects cell-state-specific enhancer landscapes of human tumor-infiltrating CD8 T cells. ***Mol Cell.*** 2023 Feb 16;83(4):622-636.e10. doi: 10.1016/j.molcel.2022.12.029. Epub 2023 Jan 18. PMID: 36657444.
CD4+ T cell	Cd4, Cd44, Cd38.	The ecto-enzyme CD38 modulates CD4T cell immunometabolic responses and participates in HIV pathogenesis. ***J Leukoc Biol.*** 2024 Sep 2;116(3):440-455. doi: 10.1093/jleuko/qiae060. PMID: 38466822. Phenotypic signatures of immune selection in HIV-1 reservoir cells. ***Nature.*** 2023 Feb;614(7947):309-317. doi: 10.1038/s41586-022-05538-8. Epub 2023 Jan 4. PMID: 36599977; PMCID: PMC9908552.
Exhausted Tcell	Cd274, Nfat5, Tox, Ctla4, Cd40	Cancer- and infection-induced T cell exhaustion are distinct. ***Nat Immunol.*** 2023 Oct;24(10):1604-1605. doi: 10.1038/s41590-023-01624-9. PMID: 37709988. Mognol GP, Spreafico R, Wong V, Exhaustion-associated regulatory regions in CD8 tumor-infiltrating T cells. ***Proc Natl Acad Sci U S A.*** 2017 Mar 28;114(13):E2776-E2785. doi: 10.1073/pnas.1620498114. Epub 2017 Mar 10. PMID: 28283662; PMCID: PMC5380094. A liver-centric help circuit revives CD8 T cells via IL-27. ***Nat Immunol.*** 2025 Jul 8. doi: 10.1038/s41590-025-02204-9. Epub ahead of print. PMID: 40629127.
Trege	Foxp3, I12ra	Regulatory T cells function in established systemic inflammation and reverse fatal

		autoimmunity. ***Nat Immunol.*** 2021 Sep;22(9):1163-1174. doi: 10.1038/s41590-021-01001-4. Epub 2021 Aug 23. PMID: 34426690; PMCID: PMC9341271.<br>IL-12 improves the anti-HCC efficacy of dendritic cells loaded with exosomes from overexpressing Rab27a tumor cells. ***Exp Cell Res.*** 2024 Jun 1;439(1):114073. doi: 10.1016/j.yexcr.2024.114073. Epub 2024 May 3. PMID: 38704079.
memory T cell	Cd27,117r, 112rb, Ccr5, Cxcr3	Signaling via a CD27-TRAF2-SHP-1 axis during naive T cell activation promotes memory-associated gene regulatory networks. ***Immunity.*** 2024 Feb 13;57(2):287-302.e12. doi: 10.1016/j.immuni.2024.01.011. PMID: 38354704; PMCID: PMC10967230.<br>Identification of a Kupffer cell subset capable of reverting the T cell dysfunction induced by hepatocellular priming. ***Immunity.*** 2021 Sep 14;54(9):2089-2100.e8. doi: 10.1016/j.immuni.2021.05.005. Epub 2021 Aug 31. PMID: 34469774; PMCID: PMC8459394.<br>Identification of a unique subset of tissue-resident memory CD4+ T cells in Crohn's disease. ***Proc Natl Acad Sci U S A.*** 2023 Jan 3;120(1):e2204269120. doi: 10.1073/pnas.2204269120. Epub 2022 Dec 27. PMID: 36574662; PMCID: PMC9910620.<br>A human memory T cell subset with stem cell-like properties. ***Nat Med.*** 2011 Sep 18;17(10):1290-7. doi: 10.1038/nm.2446. PMID: 21926977; PMCID: PMC3192229.
Naive T cell	Ccr7, Cd27	A human memory T cell subset with stem cell-like properties. ***Nat Med.*** 2011 Sep 18;17(10):1290-7. doi: 10.1038/nm.2446. PMID: 21926977; PMCID: PMC3192229.

Supplementary Table S6. Specific mechanisms and application scenarios of different liver fibrosis modeling methods.

Modeling method	Pathogenesis of the model	Key molecules	Potential areas for investigation
NASH	Immune heterogeneity	Cd45, Lgals9	Immune microenvironment-associated research
TAA	Inflammation	Il34, Csf1r	Research into inflammatory signaling pathways
BDL	Metabolic mechanisms	Tgfb1, Acvr1, Tgfbr1	Studies related to hepatic metabolic mechanisms
CCl_4	Extracellular matrix (ECM) remodeling	Igf1, Itgav, Itgb3	Studies related to the biomechanical microenvironment
